# Effects of a Nicotine Fact Sheet on Perceived Risk of Nicotine and E-Cigarettes and Intentions to Seek Information About and Use E-Cigarettes

**DOI:** 10.3390/ijerph17010131

**Published:** 2019-12-23

**Authors:** Bo Yang, Daniel Owusu, Lucy Popova

**Affiliations:** 1 Department of Communication, University of Arizona, Tucson, AZ 85721, USA; 2 School of Public Health, Georgia State University, Atlanta, GA 30302, USA

**Keywords:** nicotine education, nicotine misperception, e-cigarettes, nicotine communication

## Abstract

We examined how a nicotine fact sheet influenced smokers’ beliefs about nicotine and electronic cigarettes (e-cigarettes), a potentially less harmful alternative to conventional cigarettes. In an exploratory online experiment, 756 US adult current and recent former smokers (quit in the past 2 years) were randomized to view a nicotine fact sheet or control messages (bottled water ads). Effects of the nicotine fact sheet on perceived nicotine addictiveness, nicotine risk, comparative risk of e-cigarettes, and dual use intentions were analyzed using log-Poisson regression with robust error. Linear regression analyzed effects on perceived absolute risk and switching and information seeking intentions about e-cigarettes. Compared to control, the nicotine fact sheet doubled the probability of disagreeing that nicotine is the main cause of smoking-related disease (26.2% vs. 12.7%, RR = 2.06, 95% CI = 1.51, 2.82, *p* < 0.001). However, nearly three quarters of participants viewing the nicotine fact sheet still thought that nicotine is the main cause of smoking-related disease. The nicotine fact sheet increased smokers’ intentions to seek information about e-cigarettes (b = 0.45, 95% CI = 0.15, 0.74, *p* = 0.003). We did not find evidence suggesting unintended consequences of the nicotine fact sheet on smokers’ e-cigarettes risk perceptions or use intentions (e.g., increased dual use intentions or reduced absolute e-cigarette risk perception).

## 1. Introduction

In July 2017, the U.S. Food and Drug Administration (FDA) announced a plan for a new tobacco regulation strategy that lowers nicotine in cigarettes to minimally or nonaddictive levels [1]. This groundbreaking policy has not been tried anywhere in the world yet and may have significant positive public health impacts [2]. However, to increase the success of this regulation, it should be implemented with education about nicotine. Nicotine is the main addictive ingredient in cigarettes, but it is not the major cause of smoking-related death and disease [3]. However, people often hold the opposite belief [4,5]. In 2016, 80% of U.S. adults thought nicotine was the main cause of disease from tobacco products [4]. This misperception may mislead people into believing that low nicotine cigarettes are less harmful than current cigarettes and prevent smokers from using evidence-based smoking cessation aids [5,6] or from switching to potentially less harmful tobacco products [4,6,7,8]. These issues might be prevented by educating the public about nicotine [9].

According to theories such as the Theory of Reasoned Action [10] and the Health Belief Model [11], misperceptions about an issue could be addressed by messages counteracting the misperceptions. As a result, related behavior can also be changed. Hence, in this study, we developed a nicotine educational fact sheet and tested the effects of the fact sheet on smokers’ risk perceptions about nicotine. We also explored the effects of the fact sheet on smokers’ risk perceptions and behavioral intentions regarding electronic cigarettes (e-cigarettes), which typically contain nicotine and have been suggested as a potentially less harmful alternative for smokers who are unwilling to quit smoking [12].

## 2. Materials and Methods

### 2.1. Participants and Design

This study is a part of a larger project testing the effects of messages about e-cigarettes and nicotine. Participants were 1906 current adult smokers (smoked 100 or more cigarettes in their lifetime and currently smoking some days or every day) and recent former smokers (quit in past 2 years) recruited by a commercial research company Toluna through their online panel in summer 2018.

Toluna implemented the study as an online experiment. This exploratory online experiment first assessed baseline risk perceptions and demographic characteristics and then randomized participants to one of five message conditions stratified by smoking status and baseline quitting intentions: (1) three messages about comparative risk of e-cigarettes and cigarettes; (2) three messages in condition 1 with the FDA-mandated addiction warning added; (3) a nicotine fact sheet and three messages from condition 2, (4) a nicotine fact sheet, and (5) three bottled water ads (control messages). For the purpose of this report, we included only participants in the nicotine fact sheet condition (n = 378) and control condition (n = 378) resulting in a total of 756 participants. Differences between other conditions and the control were reported in a separate paper [13]. All subjects gave their electronic informed consent before they participated in the study. The study was conducted in accordance with the Declaration of Helsinki, and the protocol was approved by Georgia State University’s Institutional Review Board (IRB number: H18570). At the end, all participants saw a debriefing page stating that the messages were used for research only, that stopping smoking completely is the best thing they can do for their health, and were referred to smoking cessation resources.

### 2.2. Materials

The nicotine fact sheet (Box 1) was created based on literature review and discussions with tobacco control experts. It first described what nicotine is: “Nicotine is a chemical found naturally in tobacco. Nicotine is in almost all tobacco products, certain pesticides and some medicines, such as nicotine-replacement products.” It then explained that nicotine is harmful but it “is not the main cause of harm from smoking… most of the harm from smoking comes from the inhalation of tobacco smoke and other chemicals in it.” It further detailed the health harms of nicotine, “However, nicotine is not safe to use in pregnancy and can harm fetal development. Also, nicotine may cause long-term harm to adolescents’ brain development.” The fact sheet concluded with the information that nicotine is addictive but the likelihood of addiction to nicotine depends on how quickly nicotine is delivered into the bloodstream and to the brain and varies across individuals.

Box 1. **Nicotine fact sheet.**

**Nicotine**
**What is nicotine?**
Nicotine is a chemical found naturally in tobacco. Nicotine is in almost all tobacco products, certain pesticides and some medicines, such as nicotine-replacement products.
**Is nicotine harmful?**
**Yes, but it is not the main cause of harm from smoking**.
Nicotine itself is not responsible for the cancer and lung diseases that kill hundreds of thousands of Americans each year. **Most of the harm from smoking comes from the inhalation of tobacco smoke and the tar and other chemicals in it**.
Nicotine is a poison at very high doses. If you have health problems, nicotine can make them worse. Nicotine promotes cancer cell growth, reduces blood flow, and damages the reproductive system. Tobacco products and nicotine medications usually contain lower doses of nicotine, which aren’t known to cause severe health problems for most adults.
Nicotine is not safe to use in pregnancy and can harm fetal development. Also, nicotine may cause long-term harm to adolescents’ brain development.
**Is nicotine addictive?**
Yes. The main harm of nicotine is that it is addictive, which makes it so hard to stop smoking. The more quickly nicotine is delivered into the bloodstream and to the brain affects the likelihood of addiction. During smoking, nicotine enters the bloodstream rapidly, which is why people are addicted to smoking. By contrast, nicotine replacement therapies like the patch or gum deliver nicotine much more slowly and are much less likely to be addictive.
Nicotine addiction can differ among individuals, depending on their genetic profile, mental health, or personality. Social situations and environmental triggers can also affect nicotine addiction.

### 2.3. Key Measures

Key measures are shown in Table 1. Outcome variables included perceived addictiveness of nicotine and perceived risk of nicotine [5], perceived e-cigarette risk (absolute [14,15] and comparative [15,16]), and behavioral intentions (intentions to switch to e-cigarettes [15], e-cigarette and cigarette dual use intentions [15], and intentions to seek information about e-cigarettes [17,18,19,20]). Covariates included gender, age, race, education, daily vs. non-daily smoking, use of e-cigarettes (current vs. former vs. never use), quit attempt in the past 12 months, smoking identity [21], and pre-exposure perceived comparative risk of e-cigarettes, perceived addictiveness of nicotine, and perceived risk of nicotine.

### 2.4. Analysis Plan

We performed log-Poisson regression with robust error [22,23] on perceived addictiveness of nicotine, perceived risk of nicotine, and perceived comparative risk of e-cigarettes. Separate Poisson regression was performed for dual use intentions with intentions to use both e-cigarettes and cigarettes as a reference category and compared with exclusive e-cigarette use or smoking cessation intentions, respectively [24,25]. We used linear regression for perceived absolute e-cigarette risk and e-cigarette switch and information seeking intentions. For all of the analyses, we calculated both the adjusted and unadjusted effects of the nicotine fact sheet. For adjusted analyses, the models included the covariates mentioned in Section 2.3. Both adjusted and unadjusted analyses produced the same results for the effects of the nicotine fact sheet. Hence, we only discussed the unadjusted results in the following sections. Analyses were done in SPSS v24 (IBM Corp., Armonk, NY, USA) in 2019 and significance level was set at *p* < 0.05.

## 3. Results

Table 2 shows that the sample comprised 54.2% females, 70.5% Whites, and 63.4% daily smokers. Of the sample, 40.2% had high school education or less and 47% were current e-cigarette users (used e-cigarettes in past 30 days). At baseline, nearly 84% correctly understood that nicotine causes addiction but only about 14% believed that nicotine is not the major cause of smoking-related health problems. Forty-four percent believed that e-cigarettes are less harmful than cigarettes.

The nicotine fact sheet significantly increased the likelihood of people holding correct beliefs about nicotine risk: per Table 3, the proportion of people holding correct nicotine risk beliefs increased from pretest (12.7%) to posttest (26.2%) in the nicotine fact sheet condition *p* < 0.001; per Table 4, compared to the control, participants exposed to the nicotine fact sheet were more likely to correctly disagree that nicotine is the main cause of smoking-related health problems (26.2% vs. 12.7%, RR = 2.06, 95% CI = 1.51, 2.82, *p* < 0.001). There was no difference between pretest and posttest in perceived addictiveness of nicotine or perceived comparative risk of e-cigarettes in either control or nicotine fact sheet condition (Table 3). We did not find evidence suggesting a difference between the nicotine fact sheet and the control messages on perceived addictiveness of nicotine, perceived comparative or absolute risk of e-cigarettes (Table 4).

According to Table 5, exposure to the nicotine fact sheet increased intentions to seek information about e-cigarettes (M_fact sheet_ = 4.33, M_control_ = 3.88, b = 0.45, 95% CI = 0.15, 0.74, *p* = 0.003) compared to the control. There were no differences between nicotine fact sheet and control messages on intentions to switch to e-cigarettes or dual use intentions.

## 4. Discussion

In our study, most smokers already understood that nicotine is the main cause of tobacco addiction but they incorrectly believed that nicotine is the main cause of smoking-related health problems. A nicotine fact sheet helped correct this misperception—participants who saw the nicotine fact sheet were two times more likely to disagree that nicotine is the main cause of smoking-related disease. The nicotine fact sheet did not significantly change smokers’ e-cigarette risk perception or switch intentions. Together, these findings indicate that after learning about actual nicotine risk, people may be motivated to re-evaluate the risks of e-cigarettes by seeking information about e-cigarettes. We found no evidence of nicotine fact sheet increasing dual use intentions among smokers.

Although the nicotine fact sheet improved people’s understanding that nicotine is not the main substance causing smoking-related disease, among those exposed to the nicotine fact sheet, only 26.2% correctly disagreed that nicotine is the main substance causing smoking-related disease. This occurred possibly because our nicotine fact sheet also mentioned some negative health effects of nicotine. This negative information might counteract the corrective information included in the fact sheet. Alternatively, many people viewing the nicotine fact sheet held strong misbeliefs about the risk of nicotine (44.2%). According to the social judgement theory [26], it is difficult to change people’s beliefs if the beliefs are strongly endorsed. Additionally, tobacco risk communication often presents information about nicotine in tobacco products together with information about the risk of tobacco products, which can make people develop negative feelings or affect towards nicotine. Such negative affect is related to high perceived nicotine risk [27,28] and can bias processing of nicotine-related messages. In our study, this negative affect might even get reinforced due to the presence of negative information about nicotine in the fact sheet. As a result, many people did not report beliefs about the role of nicotine in smoking-related harms consistent with the fact sheet. Another potential explanation is that the nicotine risk correction information was presented without an attribution (e.g., to FDA or CDC) and participants might have attributed the information—opposite of their common beliefs—to other sources (e.g., tobacco companies) that they did not fully trust. As a result, they were not convinced by the nicotine risk correction information. Additionally, participants might have not read the nicotine fact sheet closely and could only process and remember the information more compatible with their pre-existing view towards nicotine. Very limited research to date has examined how to communicate to people about nicotine. We call for more research to be conducted on this topic to better inform tobacco regulation and communication. Particularly, future research should assess whether emphasis on combustion of cigarettes as a cause of smoking-related diseases without mentioning nicotine harms might be a better strategy to communicate about nicotine.

While the nicotine fact sheet changed perceptions of nicotine harm, it did not have an effect on perceived harm of e-cigarettes. There are several possible explanations for that. It is possible that the nicotine beliefs had more room for change compared to e-cigarette beliefs—at baseline only 12.7% of participants believed that nicotine is not the main cause of smoking-related disease while 44.2% already believed that e-cigarettes are less harmful than cigarettes. In addition, the nicotine fact sheet focused specifically on nicotine in combusted cigarettes. While many people are aware that e-cigarettes contain nicotine, a large proportion is not (for example, among UK youth 40.5% in 2013 to 34.3% in 2014 did not know if e-cigarettes contained nicotine) [29]. Thus, it seems likely that the connection between nicotine and e-cigarettes was not a very close one and the changing perceptions of harm of nicotine might not translate into lower perceived harm of e-cigarettes. Indeed, while e-cigarettes were originally designed to deliver nicotine, they are now frequently used with other substances, such as a cannabis derivative, tetrahydrocannabinol (THC), particularly by young people [30,31,32]. THC-containing e-cigarettes recently received heavy media attention due to the outbreak of nationwide e-cigarettes-related lung injury in the U.S. While our data were collected prior to the outbreak, future research on e-cigarettes should carefully distinguish between perceptions and use of e-cigarettes with nicotine vs. with other substances.

In addition, some qualitative research demonstrates that the mention of chemicals in e-cigarettes (both nicotine and other chemicals like formaldehyde) makes people view e-cigarettes as more harmful [33]. It is likely that discussing nicotine in the fact sheet made the presence of this chemical more salient for the readers, and while it decreased perceived harm of nicotine, having potential to then lower perceived harm of e-cigarettes, this might have been counterbalanced by people’s perceptions that e-cigarettes still contain nicotine, a harmful substance.

Overall, these findings suggest that a nicotine fact sheet providing balanced health information purely about nicotine might not directly influence smoker’ beliefs about e-cigarettes but may encourage smokers to learn more about e-cigarettes. The specific information smokers subsequently find about e-cigarettes may influence their decisions to use e-cigarettes and how they use the products (e.g., switch or dual use). Thus, while educating people about nicotine, health agencies should be ready to provide evidence-based information on e-cigarettes to help smokers make health-enhancing decisions when they search for information about e-cigarettes.

The study has several limitations. It prompted people to read a nicotine fact sheet, used a non-probability-based sample, and measured only cognitive responses to the nicotine fact sheet. Additionally, it only examined the effects of the nicotine fact sheet on smokers. Future studies should evaluate its effects on nonsmokers. Future studies should also evaluate how information about nicotine might affect perceptions of nicotine replacement therapy (NRT) products.

## 5. Conclusions

In conclusion, a nicotine fact sheet might be useful in fostering correct nicotine risk perceptions. We did not find evidence suggesting unintended consequences of a nicotine fact sheet on U.S. smokers’ e-cigarettes risk perceptions or use intentions (e.g., increased dual use intentions or reduced absolute e-cigarette risk perception). However, the nicotine fact sheet increased smokers’ information-seeking intentions about e-cigarettes. The eventual outcome of a nicotine fact sheet on smokers may then depend on whether information-seeking about e-cigarettes is properly guided, which warrants attention from nicotine education campaigners and tobacco product regulators.

## Figures and Tables

**Table 1:** Key measures.

Measures	Response Options	Reliability (for Scale)
**Nicotine perception**		
**Perceived addictiveness of nicotine ^a^:** To what extent, if at all, do you agree that nicotine is the main substance in tobacco that makes people become addicted to tobacco products?	1 (strongly disagree)–5 (strongly agree) + I don’t know	
**Perceived risk of nicotine ^b^:** To what extent, if at all, do you agree that nicotine in cigarettes is the substance that causes most of smoking-related health problems, such as cancer and lung disease?	1 (strongly disagree)–5 (strongly agree) + I don’t know	
**E-cigarette risk perception**		
**Perceived absolute risk:** Imagine that you just began vaping e-cigarettes every day. What do you think your chances are of having each of the following happen to you if you continue to vape e-cigarettes every day? - Lung cancer - Lung disease other than lung cancer (such as COPD ^d^ and emphysema) - Heart disease - Become addicted - Early/Premature death	0 (no chance)–6 (very good chance) + I don’t know ^c^	*α* = 0.92
**Perceived comparative risk ^e^:** Is using electronic cigarettes (vapes) less harmful, about the same, or more harmful than smoking regular cigarettes?	Three options + I don’t know	
**Behavioral intentions**		
**Intentions to switch completely to e-cigarettes:** How likely are you to switch completely from using regular cigarettes to electronic cigarettes in the next 6 months?	1 (not at all)–9 (extremely)	
**Dual use intentions:** Which of the following are you most likely to do in the next month? (Pick one) ^f^ Only smoke cigarettes Mostly smoke cigarettes and occasionally use e-cigarettes Smoke cigarettes and use e-cigarettes about the same amount Occasionally smoke cigarettes and mostly use e-cigarettes Only use e-cigarettes Not smoke cigarettes and not use e-cigarettes Other: (please write your answer)________________	Pick one option	
**Information seeking intentions:** - How interested are you in learning more about e-cigarettes in the next few months? - If you see information about e-cigarettes in the media such as newspapers, TV, radio, and the Internet in the next few months, how likely are you to pay close attention to it? - How likely are you to talk about e-cigarettes with your family and friends in the next few months? - How likely are you to talk about e-cigarettes with your doctor in the next few months?	1 (not at all)–7 (very much)	*α =* 0.94
**Covariate**		
**Smoking identity:** - Smoking is part of my self-image. - Smoking is part of “who I am.” - Smoking is a part of my personality. - Smoking is a large part of my daily life. - Others view smoking as part of my personality.	1 (strongly disagree)–10 (strongly agree)	*α =* 0.92

Notes. ^a^ The response categories “somewhat agree” and “strongly agree” were grouped together (correct knowledge about nicotine addictiveness) and compared with the combined response categories “somewhat disagree”, “strongly disagree”, “neither disagree nor agree”, and “I don’t know” (incorrect or no knowledge). ^b^ The response categories “somewhat disagree” and “strongly disagree” were grouped together (correct knowledge about nicotine health risk) and compared with the combined response categories “somewhat agree”, “strongly agree”, “neither disagree nor agree”, and “I don’t know” (incorrect or no knowledge). ^c^ The response category “I don’t know” (5%) was treated as missing value in the data analysis. ^d^ COPD = chronic obstructive pulmonary disease. ^e^ The response categories “more harmful, same, and I do not know” were grouped together and compared with the response category “less harmful.” ^f^ The response category 7 included zero cases. The response categories 2, 3, and 4 were grouped together (dual use) and compared with the response categories 5 (exclusive e-cigarette use) and 6 (cessation).

**Table 2:** Characteristics of study participants by conditions and overall.

	Nicotine Fact Sheet % (n = 378)	Control % (n = 378)	Overall % (n = 756)
**Gender**			
Male	41.0	48.4	44.7
Female	57.7	50.8	54.2
Transgender	1.1	0.8	0.9
Other	0.3	0.0	0.1
**Age**			
18–29	25.4	22.2	23.8
30–44	34.7	32.8	33.7
45–59	22.0	25.1	23.5
60+	18.0	19.8	18.9
**Race**			
White	68.8	72.2	70.5
Black or African American	16.4	15.9	16.1
American Indian or Alaska Native	1.6	2.4	2.0
Asian	5.6	3.7	4.6
Other	7.7	5.8	6.7
Education *			
Less than high school	6.3	10.1	8.2
High school	30.2	33.9	32.0
Some college	30.4	32.5	31.5
Bachelor or higher degree	33.1	23.5	28.3
**Daily smoker**			
Yes	62.7	64.0	63.4
No	37.3	36.0	36.6
**E-Cigarette use**			
Current	47.9	46.0	47.0
Former	22.2	24.1	23.1
Never	29.9	29.9	29.9
**Current cigarette smoking**			
Yes	97.1	95.8	96.4
No, former smoker	2.9	4.2	3.6
**Tried to quit in the past 12 months**			
Yes	52.9	54.8	53.8
No	47.1	45.2	46.2
**Perceived e-cigarette comparative risk at pretest**			
Less harmful	44.2	43.9	44.0
Equally harmful	37.6	38.9	38.2
More harmful	8.5	6.9	7.7
I don’t know	9.8	10.3	10.1
**Perceived addictiveness of nicotine at pretest** (“nicotine is the main substance in tobacco that makes people become addicted to tobacco products”)			
Strongly agree	62.2	60.1	61.1
Somewhat agree	21.7	23.3	22.5
Neither agree nor disagree	8.7	8.7	8.7
Somewhat disagree	2.4	4.0	3.2
Strongly disagree	2.1	2.6	2.4
I don’t know	2.9	1.3	2.1
**Perceived risk of nicotine at pretest** (“nicotine in cigarettes is the substance that causes most of smoking-related health problems”)			
Strongly agree	44.2	37.3	40.7
Somewhat agree	26.5	26.7	26.6
Neither agree nor disagree	14.3	16.9	15.6
Somewhat disagree	7.1	11.4	9.3
Strongly disagree	5.6	4.0	4.8
I don’t know	2.4	3.7	3.0

Notes. There were no significant differences among the conditions on any of the variables except education χ^2^(3) = 10.30, *p* = 0.02. * *p* < 0.05.

**Table 3:** Descriptive statistics of key outcomes.

Outcomes		Nicotine Fact Sheet (n = 378)	Control (n = 378)
Perceived addictiveness of nicotine	Pre vs. posttest correct % (n)	83.9 (317) vs. 82.3 (311)	83.3 (315) vs. 85.7 (324)
Perceived risk of nicotine	Pre vs. posttest correct % (n)	12.7 (48) vs. 26.2 (99) ***	15.3 (58) vs. 12.7 (48)
Perceived e-cigarette comparative risk	Pre vs. posttest less harmful % (n)	44.2 (167) vs. 45 (170)	43.9 (166) vs. 42.3 (160)
Dual use intentions	Cessation % (n)	7.4 (28)	8.7 (33)
	Exclusive e-cigarette use % (n)	11.9 (45)	11.9 (45)
	Continued cigarette use % (n)	42.6 (161)	42.3 (160)
	Dual use %	38.1 (144)	37 (140)
Perceived e-cigarette absolute risk ^a^	Mean (SD)	3.68 (1.68)	3.72 (1.53)
E-cigarette switch intentions	Mean (SD)	4.81 (2.97)	4.41 (2.96)
E-cigarette information seeking intentions	Mean (SD)	4.33 (2.04)	3.88 (2.04)

Notes. Correct perceived addictiveness of nicotine = strongly agree or somewhat agree with the statement that nicotine is the main substance in tobacco that makes people become addicted to tobacco products; correct perceived risk of nicotine = strongly disagree or somewhat disagree with the statement that nicotine in cigarettes is the substance that causes most of the smoking related health problems, such as cancer and lung disease. Pretest and posttest difference for each outcome was examined through McNemar’s test. ^a^ Due to missing value in the data analysis, the displayed means are based on 363 cases in nicotine fact sheet condition and 355 cases in the control condition. Boldface indicates statistical significance for paired comparisons (* *p* < 0.05, ** *p* < 0.01, *** *p* < 0.001).

**Table 4:** Effects of a nicotine fact sheet on risk perceptions.

Predictor	Perceived Addictiveness of Nicotine (Correct vs. Incorrect or Don’t Know), RR (95% CI) ^a,c^	Perceived Risk of Nicotine (Correct vs. Incorrect or Don’t Know), RR (95% CI) ^a,c^	Perceived E-Cigarette Comparative Risk (Less vs. More or Equally Harmful or Don’t Know), RR (95% CI) ^a,c^	Perceived E-Cigarette Absolute Risk, b (95% CI) ^b,d^
	Unadjusted			
Nicotine fact sheet (vs. control)	0.96 (0.90, 1.02)	2.06 *** **(1.51, 2.82)**	1.06 (0.90, 1.25)	−0.04 (−0.27, 0.20)
	Adjusted			
Nicotine fact sheet (vs. control)	0.96 (0.91, 1.02)	2.29 *** **(1.76, 2.98)**	1.07 (0.95, 1.20)	−0.08 (−0.30, 0.15)

Notes. Correct perceived addictiveness of nicotine = strongly agree or somewhat agree with the statement that nicotine is the main substance in tobacco that makes people become addicted to tobacco products; correct perceived risk of nicotine = strongly disagree or somewhat disagree with the statement that nicotine in cigarettes is the substance that causes most of the smoking related health problems, such as cancer and lung disease. Adjusted analyses controlled for gender, age, race, education, daily use of cigarettes, use of e-cigarettes (current vs. former vs. never use), quit attempt in the past 12 months, smoking identity, and pre-exposure perceived comparative risk of e-cigarettes, perceived addictiveness of nicotine, and perceived risk of nicotine. ^a^ Log-Poisson model with robust error. ^b^ Linear regression. ^c^ n = 756. ^d^ n = 718. RR = relative risk; b = unstandardized regression coefficient. Boldface indicates statistical significance (* *p* < 0.05, ** *p* < 0.01, *** *p* < 0.001).

**Table 5:** Effects of a nicotine fact sheet on behavioral intentions.

Predictor	E-Cigarette Switch Intentions, b (95% CI) ^a,c^	E-Cigarette Information Seeking Intentions, b (95% CI) ^a,c^	Cessation (vs. Dual Use) Intentions, RR (95% CI) ^b,d^	Exclusive E-Cigarette Use (vs. Dual Use) Intentions, RR (95% CI) ^b,e^
	Unadjusted			
Nicotine fact sheet (vs. control)	0.40 (−0.02, 0.82)	0.45 ** **(0.15, 0.74)**	0.98 (0.91, 1.05)	0.99 (0.88, 1.12)
	Adjusted			
Nicotine fact sheet (vs. control)	0.26 (−0.06, 0.59)	0.35** **(0.12, 0.58)**	1.00 (0.94, 1.06)	1.02 (0.91, 1.15)

Notes. Adjusted analyses controlled for gender, age, race, education, daily use of cigarettes, use of e-cigarettes (current vs. former vs. never use), quit attempt in the past 12 months, smoking identity, and pre-exposure perceived comparative risk of e-cigarettes, perceived addictiveness of nicotine, and perceived risk of nicotine. ^a^ Linear regression. ^b^ Log-Poisson model with robust error. ^c^ n = 753. ^d^ n = 345. ^e^ n = 374. RR = relative risk; b = unstandardized regression coefficient. Boldface indicates statistical significance (* *p* < 0.05, ** *p* < 0.01, ***p < 0.001).
